# A population study of prolonged grief in refugees

**DOI:** 10.1017/S2045796019000386

**Published:** 2019-08-19

**Authors:** R. A. Bryant, B. Edwards, M. Creamer, M. O'Donnell, D. Forbes, K. L. Felmingham, D. Silove, Z. Steel, A. C. McFarlane, M. van Hooff, A. Nickerson, D. Hadzi-Pavlovic

**Affiliations:** 1 School of Psychology, University of New South Wales, Sydney, Australia; 2 Centre for Social Research and Methods, Australian National University, Canberra, Australia; 3 Phoenix Australia, University of Melbourne, Melbourne, Australia; 4 School of Psychological Sciences, University of Melbourne, Melbourne, Australia; 5 School of Psychiatry, University of New South Wales, Sydney, Australia; 6 Centre for Traumatic Stress Studies, University of Adelaide, Adelaide, Australia

**Keywords:** Bereavement, prevalence, prolonged grief disorder, refugees

## Abstract

**Aims:**

Despite the frequency that refugees suffer bereavement, there is a dearth of research into the prevalence and predictors of problematic grief reactions in refugees. To address this gap, this study reports a nationally representative population-based study of refugees to determine the prevalence of probable prolonged grief disorder (PGD) and its associated problems.

**Methods:**

This study recruited participants from the Building a New Life in Australia (BNLA) prospective cohort study of refugees admitted to Australia between October 2013 and February 2014. The current data were collected in 2015–2016, and comprised 1767 adults, as well as 411 children of the adult respondents. Adult refugees were assessed for trauma history, post-migration difficulties, probable PGD, post-traumatic stress disorder (PTSD) and mental illness. Children were administered the Strengths and Difficulties Questionnaire.

**Results:**

In this cohort, 38.1% of refugees reported bereavement, of whom 15.8% reported probable PGD; this represents 6.0% of the entire cohort. Probable PGD was associated with a greater likelihood of mental illness, probable PTSD, severe mental illness, currently unemployed and reported disability. Children of refugees with probable PGD reported more psychological difficulties than those whose parents did not have probable PGD. Probable PGD was also associated with the history of imprisonment, torture and separation from family. Only 56.3% of refugees with probable PGD had received psychological assistance.

**Conclusions:**

Bereavement and probable PGD appear highly prevalent in refugees, and PGD seems to be associated with disability in the refugees and psychological problems in their children. The low rate of access to mental health assistance for these refugees highlights that there is a need to address this issue in refugee populations.

## Introduction

The current refugee crisis has resulted in over 22 million refugees across the globe, and over 40 million internally displaced persons (UNHCR, 2017). Refugees experience elevated rates of mental disorder, including depression, post-traumatic stress disorder (PTSD) and anxiety (Steel *et al*., 2009). Considering the widespread exposure to war, civil conflict and family separation, it is surprising that more attention has not focused on grief in refugees because they can be vulnerable to much bereavement resulting from the cumulative adversity they and their loved one's experience (Tay *et al*., 2016). Prolonged grief disorder (PGD) is a new diagnostic construct in ICD-11, and is described as persistent yearning for the deceased and associated emotional pain, difficulty in accepting the death, a sense of meaninglessness, bitterness about the death and difficulty in engaging in new activities(World Health Organization, 2018). A major rationale for its recognition in ICD-11 is that PGD is associated with marked mental, social and physical impairment (Shear, 2015), and is reported in approximately 7% of bereaved people (Maciejewski *et al*., 2016). Rates of probable PGD in refugees have varied between 8 and 54% (Craig *et al*., 2008; Schaal *et al*., 2010), but these have been in small, clinical or non-representative studies. Accordingly, these rates do not provide accurate estimates of the occurrence of PGD in refugees. The goal of this study was to conduct a population-based study of refugees to determine the prevalence, predictors and costs of probable PGD in a large, nationally representative sample of refugees. We included the analysis of potential manifestations of mental health conditions commonly reported in refugees. Specifically, we measured (a) the association of PGD with probable PTSD because of the increased risk for PTSD resulting from repeated exposure to traumatic events (Steel *et al*., 2009), (b) psychological distress because of the ongoing stressors experienced by refugees (Li *et al*., 2016) and (c) self-reported functional disability because of the potential impacts of marked psychological distress on functioning. Additionally, we assessed the psychological status of the children of the refugee respondents because of evidence that refugee parental/caregiver mental health can impact children's mental health (Lambert *et al*., 2014). No prior studies have specifically assessed refugee childhood mental health in the context of caregivers' PGD, and so we additionally questioned the respondents' children about their psychological well-being.

## Method

### Participants

The analysis is based on the Building a New Life in Australia (BNLA) study undertaken by the Australian Government Department of Social Services (DSS) (Edwards *et al*., 2018). The BNLA is a longitudinal cohort study of refugees. The study was designed to recruit 1500 migration units, from specified locations, and in specified proportions according to offshore (refugee) or onshore (asylum seeker) status, and visa category. Eligibility criteria for the BNLA included: (1) being the principal or secondary applicant (i.e. the refugee applicant within a family unit) for a humanitarian visa awarded between May 2013 to December 2013; and (2) being 18 years and older. Eligible principal applicants consented to participate, as well as for other adult members of their migrating unit to be invited to participate. These secondary applicants were then invited to participate in the BNLA study if they were residing with the principal applicant. At Wave 1, there were 4035 migration units identified as potential participants of whom 2039 (50%) were contacted, of whom in turn 1509 principal applicants (equivalent to migration units [37% of potential participants]) agreed to be interviewed (along with 890 adult/adolescent secondary applicants). Wave 1 interviews were conducted between October 2013 and February 2014. Subsequent waves were conducted annually, with Wave 3 data being collected between October 2015 and February 2016. BNLA participants were recruited from 11 sites across Australia encompassing major cities and regional areas that reflected concentrations of eligible refugees across visa classes.

The Wave 3 data also included the assessment of participants' children. The primary caregiver (not necessarily the primary refugee applicant) was invited to complete a child module with respect to up to two children in their care; if there were more than two eligible children in a household, two children were randomly selected. Two principles were applied to sampling children: (1) up to two children from each family could participate; and (2) older children (aged between 11 and 17 years) were preferentially selected over younger children because the former were able to provide self-report responses in addition to parental reports. Based on these criteria, youth were selected by randomly selecting up to two children aged 11–17 years old in each migrating unit. In households where there were two or more children aged 11–17 years old, two randomly selected children in this age group were selected. The current child data focus on the child self-reports.

Interviews were conducted using computer-assisted personal interview (CAPI) and computer-assisted self-interview (CASI). CAPI enabled field interviewers to ask questions displayed on the screen and enter responses from the participant directly into a computer. CASI allowed for participants to respond privately to self-report questions using a computer interface (assistance provided when needed). Interview questions and associated written material were translated from English into nine languages including Arabic, Burmese, Dari, Hazaragi, Persian, Chin Haka, Nepali, Swahili and Tamil. In most cases, participants were matched with an interviewer who was a native speaker of their respective language; however, few participants opted for translated/interpreted interviews. The Wave 3 data were collected after a residency period of 2–3 years in Australia (for 87% of the sample). In addition to the previous waves, Wave 3 included an assessment of bereavement and probable PGD. At Wave 3, there were 1767 adult refugees interviewed, and 411 children provided self-report data.

### Measures

#### Demographic interview

The face-to-face interview assessed a broad range of demographic and household factors.

#### Prolonged grief disorder

Bereavement was assessed by asking respondents if someone close to them had died. If respondents answered in the affirmative, they were asked the following questions regarding a death that occurred at least 6 months earlier. To assess probable PGD, we employed a screening measure for PGD that was used to assess probable PGD following Hurricane Katrina (Shear *et al*., 2011). Four questions were asked about grief in the past 30 days associated with their bereavement: (1) *How often have you found yourself or yearning for the people who died?*, (2) *Have you had trouble accepting the death?*, (3) *Do you feel bitter over their death?*, (4) *Do you feel that life is unfulfilling, empty, or meaningless since their death?*. Each response was answered on a five-point scale (1 = *not at all*, 5 = *several times a day/overwhelmingly*). The initial use of this measure following Hurricane Katrina found that a principal axis factor analysis yielded one meaningful factor with an unrotated eigenvalue of 2.7, and factor loadings of at least 0.77 for all items (Shear *et al*., 2011). A final question asked that each of these symptoms had been happening for at least 6 months. We scored these responses to identify probable PGD in a way that is consistent with the ICD-11 PGD criteria. Specifically, the definition of probable PGD was operationalised as the presence of yearning for the deceased at least every day, and at least two of the three other potential PGD symptoms being endorsed at least at moderate levels.

#### Post-traumatic Stress Disorder-8

The Posttraumatic Stress Disorder-8 items (PTSD-8) (Hansen *et al*., 2010) is an eight-item self-reported screening measure for probable PTSD that was used to assess the caregivers' PTSD symptoms. The PTSD-8 is designed for assessing trauma symptoms in refugee populations, and assesses four intrusion, two avoidance and two hypervigilance items. Participants rate the symptoms on a four-point Likert scale (1  =  *not at all*, 4  =  *most of the time*) reporting how much the symptoms bothered them in the past week. Probable diagnosis of PTSD is met if at least one symptom from each of the three PTSD-8 subscales has an item score of 3 or 4 (indicating the symptom is present ‘sometimes’ or ‘most of the time’ for at least one item in each subscale) (Hansen *et al*., 2010). This measure was administered at all three waves and had an internal consistency (Cronbach's *α*) of 0.91–0.92 across waves.

#### Severe mental illness

The Kessler Screening Scale for Psychological Distress (K6; Kessler *et al*., 2010) was used to index severe mental illness in the previous 4 weeks. Participants rate each symptom of depression or anxiety on a five-point Likert scale (0  =  *none of the time*, 4  =  *all of the time*), with scores ⩾19 classified as severe mental illness. The K6 has been used cross-culturally across many studies in numerous languages, with scores ⩾19 classified as severe mental illness (Australian Bureau of Statistics, 2012). In the present study, the internal consistency of the K6 was 0.91.

#### Trauma history

To assess the extent of exposure to traumatic events, at Wave 3, caregivers were administered a modified version of the Harvard Trauma Questionnaire Trauma Events Module (Mollica *et al*., 1992) that indexed ten potentially traumatic events. This measure comprises events that are relevant to refugee experience (e.g. combat exposure, imprisonment, murder/disappearance of family members) and has been widely used with refugee populations (Hollifield *et al*., 2002).

#### Post-migration stressors

The BNLA indexed post-migration stressors commonly experienced by refugees in resettlement period. Specifically, respondents were asked to dichotomously indicate if any of the ten life events caused them stress; events included work, housing, finances, family safety, discrimination, school/study, caring for family, conflict with neighbors, language barriers or acculturation.

#### Strengths and Difficulties Questionnaire

The Strengths and Difficulties Questionnaire (SDQ; Goodman, 2001) was used to assess the children's psychological problems. The SDQ comprises 25 items measuring behavioural, emotional and social problems that children can report on a three-point Likert scale (0  =  *not true*, 2  =  *certainly true*). It comprises of five subscales: conduct problems, hyperactivity, emotional symptoms, peer problems and prosocial behaviour (this analysis focuses on the four difficulties subscales). In the current study, all items (except the prosocial items) were summed to establish a total difficulties score (range 0–40). The SDQ possesses good internal consistency for screening child psychiatric disorders (Goodman, 2001), and is a widely used measure of adjustment among refugee populations and has comparable factor structures across ethnic groups (Richter *et al*., 2011).

#### Data analysis

All analyses incorporated the Wave 3 participant survey weight, which was based on visa subclass, capital city, age and country of birth information (Australian Government, 2017). We initially report the frequencies of refugees with and without probable PGD, along with *χ*^2^analyses of main associated outcomes that include demographic characteristics, psychological comorbidities and help-seeking. We then report adjusted odds ratios of the likelihood of developing probable PGD in relation to exposure to different types of traumatic events and current stressors. These calculations adjusted for age, gender, marital status and country of origin to determine the impact of adverse events on the likelihood of probable PGD beyond the influence of these factors. To determine the association between childhood mental health and caregiver PGD, proportions of children with psychological difficulties on each of the SDQ subscales were compared in terms of children whose caregiver had and did not have PGD. All child data were related to the primary caregiver data because the primary caregiver provided the child data; accordingly, all analyses of child data included a cluster effect of the migration unit.

## Results

### Participant characteristics

The majority of adult participants were male (785, 61.7%), and most reported being married or having a partner (866, 68.1%). In terms of country of origin, after excluding 26 (2.0%) with confidential data, participants came from Iraq or Afghanistan (684, 54.9%); Bhutan or Myanmar (213, 17.1%), Iran (100, 8.1%), Libya/Syria/Egypt (86, 6.9%), Pakistan (76, 6.1%), sub-Sahara Africa (53, 4.2%) and Sri Lanka/India (35, 2.8%). In this sample, 502 (39.4%) respondents reported the death of someone close to them. Calculating probable PGD on the basis of the ICD-11 criteria, 75 satisfied our criteria for probable PGD; this represents 15.8% of those who were bereaved, and 6.0% of the entire cohort. Subsequent analyses focus on these criteria for probable PGD.

In terms of the children respondents, the proportion of females (46.6%) and males (53.4%) was similar (*p*  =  0.1063). The mean age of the children was 11.6 years (s.d.  =  3.9) and did not differ between females and males (*M*  =  11.5 and 11.6, respectively; *p*  =  0.6503). There were 29.3% of children below the age of 10; 25.7% aged 10–12; 27.7% aged 13–15; and 17.3% aged 16 and over, with highly similar percentages for both sexes (*p*  = 0.9140). Caregivers reported that 24.6% of children (of all ages) had experienced either a trauma or a threat to their life or both, and 25.4% of children (aged 11 or more) self-reported such experiences.

### Correlates with probable prolonged grief disorder

Table 1 presents the participant characteristics of those who did and did not meet the more stringent criteria for probable PGD. Refugees with probable PGD were more likely to be female and not married. In terms of problems associated with persistent grief, those with probable PGD were more likely to be older, and to report severe mental illness, probable PTSD, be unemployed and disability. Only 56.3% of those with probable PGD reported ever receiving psychological assistance in Australia (but of those, 91.4% did so in the last 12 months), compared to 35.0% of those without PGD (80.2% in the last 12 months).

**Table 1.** Participant characteristics

Participants (*N* = 1245 [1302])							
		PGD (*n* = 75 [99])		No PGD (*n* = 1171 [1203])			
	AN	*N*	%	*N*	%	*F*	*p*
Gender, female	1245	38	50.8	435	37.2	6.37	0.0118
Married	1244	45	60.9	799	68.3	1.73	0.1884
Disability	1242	48	64.0	340	29.1	42.64	<0.0001
Severe mental illness	1223	30	40.5	182	15.8	31.91	<0.0001
PTSD	1209	48	64.8	318	28.00	51.26	<0.0001
Employed	1262	3	5.0	336	27.8	19.69	<0.0001
Received psychological help in Australia	1232	42	56.3	405	35.0	13.33	0.0003
Received psychological help last 6 months^a^	446	38	91.5	325	80.2	3.97	0.0469
Years since arrival	1246					1.25	0.2832
1–2		2	3.6	42	3.5		
2–3		62	73.8	864	74.4		
3+		11	22.6	264	22.1		
		*M*	S.E.	*M*	S.E.	*F*	*p*
Age, years	75/1171	45.89	1.57	38.78	0.41	19.16	<0.0001

AN, applicable *N* (weighted), i.e. not coded as ‘not applicable’ (in most cases) or otherwise missing. Totals are weighted totals rounded to the nearest integer, except for totals in brackets which are observed totals. As reported by *Stata*, *F* for categorical data is a design-based *F*, and *F* for continuous data is an adjusted Wald test.

a Only asked of those who have received help in Australia.

Table 2 presents the adjusted odds ratios of having probable PGD after controlling for age, gender, marital status and time since arriving in Australia. Refugees were markedly more likely to develop probable PGD if they had suffered murder or disappearance of a family member (AOR 3.16; 95% CI 1.91–5.23), experienced torture (AOR 2.05; 95% CI 1.13–3.71) or experienced combat (AOR 1.69; 95% CI 1.04–2.74). Further, those with probable PGD had experienced more personal traumatic events and also had more post-migration stressors.

**Table 2.** Adjusted odds ratios and 95% confidence intervals for associations between prolonged grief disorder and pre-migration and post-migration stressors (controlling for age, gender, marital status and time since arriving in Australia)

Participants (*N* = 1245 [1302])							
	PGD (*n* = 75 [99])		No PGD (*n* = 1171 [1203])				
	*N*	%	*N*	%	AOR	95% CI	*p*
Combat exposure	43	62.2	417	48.5	1.69	1.04–2.74	0.0348
Extreme living conditions	34	49.0	410	47.7	0.97	0.61–1.54	0.8921
Serious injury	20	28.4	169	19.7	1.60	0.95–2.67	0.0758
Imprisonment	21	30.6	189	22.0	1.61	0.96–2.68	0.0721
Separation from family	16	23.1	163	19.0	1.44	0.81–2.56	0.2111
Murder/disappearance of family	23	32.8	156	18.1	2.15	1.26–3.66	0.0052
Murder/disappearance of a stranger	24	34.1	218	25.3	1.49	0.87–2.55	0.1441
Torture	19	27.7	139	16.1	2.11	1.19–3.74	0.0105
Gender-based violence	14	20.4	130	15.1	1.34	0.67–2.67	0.4057
	*N*	*M* (S.E.)	*N*	*M* (S.E.)			
# of types of personal trauma	69	3.73 (0.20)	860	2.75 (0.07)	1.21	1.11–1.32	<0.0001
# of post-migration stressors	71	2.92(0.23)	1133	2.46 (0.07)	1.12	0.99–1.27	0.0629

For the trauma-exposure questions, after excluding ‘not applicable’ and missing, the weighted *N*  =  930 (69 ‘PGD’ and 860 ‘No PGD’); for the ‘stressors’ variable the weighted *N*  =  1204 (71 ‘PGD’ and 1133 ‘No PGD’).

In terms of post-migration stressors, Table 3 indicates that refugees with probable PGD were more likely to have experienced discrimination (AOR 2.38; 95% CI 1.06–5.34), and also more likely to be in receipt of government benefits (AOR 3.71; 95% CI 1.32–10.38).

**Table 3.** Adjusted odds ratios and 95% confidence intervals for associations between prolonged grief disorder and post-migration stressors (controlling for age, gender, marital status and time since arriving in Australia)

Participants (*N* = 1245^a^ [unweighted: 1302])							
	PGD (*n* = 75 [99])		No PGD (*n* = 1171 [1203])				
	*N*	%	*N*	%	AOR	95% CI	*p*
Family waiting to come to AU	35	47.3	642	55.2	0.76	0.48–1.20	0.2399
Does not speak English well	48	63.8	634	54.8	0.89	0.53–1.48	0.6506
Look for paid work in last 12 months	15	20.5	448	39.2	0.57	0.32–1.03	0.0634
Receives government benefits	70	94.0	860	74.1	3.71	1.32–10.38	0.0124
No friends in Australia	6	8.2	65	5.7	1.29	0.41–4.08	0.6600
Has experienced discrimination	9	12.7	86	7.4	2.38	1.06–5.34	0.0361
Does not trust neighbourhood	11	17.7	199	19.4	0.91	0.47–1.75	0.7751
Does not trust Australian community	16	29.3	178	17.5	1.81	0.89–3.69	0.1021
Does not trust government	6	8.5	81	7.4	1.48	0.48–4.53	0.4938
Does not trust health professionals	7	9.4	75	6.8	1.52	0.55–4.18	0.4178
Support from ethnic community^b^	40	54.5	589	51.3	1.01	0.76–1.33	0.9674
Support from religious community^b^	42	57.3	617	53.9	1.04	0.78–1.39	0.7900
Support from other groups^b^	52	70.8	725	63.8	1.19	0.85–1.65	0.3063
Feels welcome in Australia^c^	9	11.6	137	11.8	0.97	0.68–1.37	0.8453
Sense of belonging in Australia^d^	15	19.4	195	16.9	1.07	0.82–1.41	0.6075

a The is maximum *N*: sample sizes below are reduced by missing values.

b Three-point scale (‘yes’, ‘sometimes’, ‘no’).

c Four-point scale (‘always’ to ‘never’; percentage for ‘sometimes/never’).

d Five-point scale (‘always’ to ‘never’; percentage for ‘sometimes/hardly/never’).

Table 4 indicates that children of respondents with probable PGD had markedly more severe emotional problems on the SDQ (*t*_338_ = 3.23, *p* = 0.0013). The groups did not differ in relation to conduct problems, hyperactivity or peer problems.

**Table 4.** Means on primary caregiver-reported SDQ subscales

	PGD (*N* = 26) [40 children]		No PGD (*N* = 313) [498 children]			
SDQ subscales	*M*	S.E.	*M*	S.E.	*t*	*p*
Emotional problems	3.10	0.29	2.10	0.12	3.23	0.0013
Conduct disorders	1.53	0.27	1.41	0.11	0.44	0.6639
Hyperactivity	3.26	0.38	3.00	0.16	0.57	0.5719
Peer problems	2.48	0.22	2.41	0.09	0.77	0.7691

The 339 primary caregivers are 95.8% of primary caregivers, and the 538 children are 94.6% of children.

## Discussion

In this sample of 1245 adult refugees, 474 (38.1%) reported experiencing bereavement. Of those who were bereaved, 75 (15.8%) respondents indicated they experienced probable PGD; this represents 6.0% of the entire cohort. The observed prevalence of probable PGD is markedly lower than reported in most prior estimates of PGD in refugees, which have reported PGD in 8–54% (Craig *et al*., 2008; Schaal *et al*., 2010). These studies comprised relatively small samples and were often recruited from treatment services. The current study comprises a nationally representative sample and therefore probably reflects a more accurate index of the prevalence of PGD in refugees. In this sense, this finding accords with meta-analytic evidence that rates of psychological disorder in refugees tend to be lower in larger and more representative studies (Steel *et al*., 2009). It is noteworthy that the prevalence of PGD in these refugees was nearly twice that of a previous nationally representative sample that found PGD in the general community was 3.7% (Kersting *et al*., 2011).

The presence of probable PGD was strongly associated with psychological comorbidity and impairment, including PTSD, severe mental illness (as measured by the K6), reported disability, not being employed and difficulty trusting others in the community. There is much evidence of high levels of comorbidity with PGD, including anxiety, depression and PTSD (Melhem *et al*., 2001; Simon *et al*., 2007). There is also much evidence that PGD is associated with work and social functioning impairments (Prigerson *et al*., 1997; Bonanno *et al*., 2007). In the context of concerns about refugees adjusting to their new lives in the host country, these patterns are noteworthy because they highlight that persistent grief problems are associated with compounded psychological and social challenges that are likely to interfere with refugees' capacities to optimally adjust to their new settings.

The likelihood of developing probable PGD was significantly heightened by exposure to a wide range of trauma and non-traumatic stressors. Not surprisingly, murder or disappearance of family members and separation from family were two of the traumatic events that had the strongest association with probable PGD. Violent death is one of the strongest predictors of PGD (Neria *et al*., 2007), which may be attributed to the suddenness of the loss, concerns about how the person died or possibly associated PTSD. There is increasing evidence that mental health problems of refugees are compounded by the level of stressors in the post-migration period (Li *et al*., 2016). The observation that probable PGD was associated with more post-migration difficulties accords with this evidence; it is difficult to disentangle the directionality of this association because whereas these stressors may compound grief responses, persistent grief may also contribute to stressors.

There was a statistically significant association between probable PGD and worse emotional difficulties in children. There is considerable evidence that parental mental health is associated with the psychological health of their children, with most attention given to the relationship between Holocaust survivors' mental health and the psychological well-being of their children (van Ijzendoorn *et al*., 2003). There is also much evidence of refugees' mental health potentially impacting on their children's psychological health, with one meta-analysis suggesting parental PTSD had a moderate impact (*r*  = 0.35) on children's psychological problems (Lambert *et al*., 2014). This association could be attributed to several factors. There is evidence that adult mental health problems negatively impact parenting behaviour, which in turn can influence children's psychological well-being (McLeod *et al*., 2007). It is also possible that parental PGD directly impacted child mental health via modelling or adverse effects of negative mood by the parent. We emphasise that the findings regarding the children's psychological difficulties are interpreted cautiously because the number of children of caregivers with probable PGD was small, and so definitive inferences about the relationship between refugees' PGD and their children's mental health awaits replication with larger sample sizes.

A concerning finding in this study was that only 56.3% of those with probable PGD had received psychological assistance. Help-seeking among people with PGD is often low, with one study finding that only 43% of those with PGD sought mental health assistance (Lichtenthal *et al*., 2011). Moreover, refugees display very low levels of mental health service utilisation (Kinzie, 2006). Sub-optimal help-seeking in bereaved refugees may be due to stigma about attending mental health services (Papadopoulos *et al*., 2003), ignorance of appropriate referral opportunities (Bartolomei *et al*., 2016) or avoidance of confronting emotional discomfort associated with their grief (Lichtenthal *et al*., 2011). The current finding highlights the need to overcome the apparent barriers for refugees with PGD to access mental health services. Interestingly, 35% of respondents without PGD had received psychological help in Australia. This could be attributed to (a) refugees receiving help for psychological conditions other than PGD (e.g. PTSD, depression), (b) having been successfully treated for PGD that was resolved by the time of this assessment or (c) provision of psychological supports for bereaved refugees without regard to their PGD status.

These observations are qualified by several methodological limitations. First, probable PGD was assessed with an abridged measure of PGD; although the screening measure complies with the ICD-11 symptom definition of PGD, it did not include an index of grief-related impairment which is one of the ICD-11 requirements; greater confidence in the observed rates of probable PGD would be achieved with a complete diagnostic measure of PGD. Relatedly, we recognise that cultural variations exist in the conceptualisation of prolonged grief, and our measure may not have optimally accommodated for these differences in the different ethnicities represented in this cohort. Second, this assessment of bereavement responses was embedded in a large study of many factors relevant to refugee adjustment, and accordingly many factors directly relevant to the development of PGD could not be adequately assessed; this precluded investigation into many potential predictors, such as the type of death, relationship to the deceased, attachment issues and mourning rituals. Third, this sample focused on refugees who were granted permanent protection visa prior to arrival in Australia and did not include current asylum applicants; this sampling resulted in this cohort not including refugees who may have some of the adverse experiences associated with seeking asylum, such as being in a detention camp. As this sample comprised refugees who had been granted humanitarian visas, it is also possible they were more trauma-exposed than some other refugee groups. Fourth, most of the sample were accompanied by family members, which may moderate the extent to which we observed problematic grief reactions because being separated from family members may exacerbate separation anxieties, which could compound grief responses. Fifth, respondents were able to respond to questions using self- or interview-administered formats, and this may have introduced some variability; we were not able to differentiate between these formats because respondents chose the format for each question depending on their literacy and confidentiality needs in response to specific questions.

In summary, this report highlights the elevated risk that refugees have for problematic grief reactions. This risk is not surprisingly associated with their exposure to traumatic loss that is often associated with war or conflict, but is also apparently heightened by the level of ongoing stressors in the refugees' postmigration setting. A significant proportion of refugees do not seek assistance for their PGD, and this points to the need for removing barriers to accessing appropriate care. In the context of strong evidence for the efficacy of grief-focused therapies in alleviating PGD symptoms (Bryant *et al*., 2014; Shear *et al*., 2016), there are good reasons to provide more targeted programmes to address the persistent grief problems experienced by refugees.

## Data

The data that support the findings of this study are available from the Australian Institute of Family Studies but restrictions apply to the availability of these data, which were used under licence for the current study, and so are not publicly available. However, data are available from the authors upon reasonable request and with the permission of AIFS. The data can be requested from http://www3.aifs.gov.au/bnla/index.html.
